# A cluster analysis of patients with axial spondyloarthritis using tumour necrosis factor alpha inhibitors based on clinical characteristics

**DOI:** 10.1186/s13075-021-02647-z

**Published:** 2021-11-15

**Authors:** Seulkee Lee, Seonyoung Kang, Yeonghee Eun, Hong-Hee Won, Hyungjin Kim, Hoon-Suk Cha, Eun-Mi Koh, Jaejoon Lee

**Affiliations:** 1 Department of Medicine, Samsung Medical Center, Sungkyunkwan University School of Medicine, 81 Irwon-ro, Gangnam-gu, Seoul, 06351 Republic of Korea; 2 Samsung Advanced Institute for Health Sciences & Technology (SAIHST), Sungkyunkwan University, Samsung Medical Center, Seoul, Republic of Korea

**Keywords:** Spondyloarthritis, TNF, Cluster analysis, Drug survival

## Abstract

**Background:**

This study aimed to classify the distinct group of patients with axial spondyloarthritis (SpA) on tumour necrosis factor alpha inhibitors (TNFi) according to the baseline characteristics using a clustering algorithm.

**Methods:**

The clinical characteristics and demographic data of patients with axial SpA included in the Korean College of Rheumatology Biologics and Targeted Therapy registry were investigated. The patterns of disease manifestations were examined using divisive hierarchical cluster analysis. After clustering, we compared the clinical characteristics of patients and the drug survival of TNFi between the classified groups.

**Results:**

A total of 1042 patients were analysed. The cluster analysis classified patients into two groups: axial group predominantly showing isolated axial manifestations (*n* = 828) and extra-axial group more frequently showing extra-axial symptoms (*n* = 214). Almost all extra-axial symptoms (peripheral arthritis, enthesitis, uveitis, and psoriasis) were more frequently observed in the extra-axial group than in the axial group. Moreover, patients in the extra-axial group had shorter disease duration, later disease onset, and higher disease activity than those in the axial group. The disease activity was comparable between the two groups after 1 year of treatment with TNFi. Interestingly, the extra-axial group had a lower drug survival with TNFi than the axial group (*p* = 0.001).

**Conclusions:**

Cluster analysis of patients with axial SpA using TNFi classified two distinct clinical phenotypes. These clusters had different TNFi drug survival, clinical characteristics, and disease activity.

**Supplementary Information:**

The online version contains supplementary material available at 10.1186/s13075-021-02647-z.

## Background

Axial spondyloarthritis (SpA), comprising ankylosing spondylitis (AS) and non-radiographic axial SpA, is the main form of chronic inflammatory arthritis affecting the axial skeleton [1, 2]. Axial SpA has multiple phenotypes which varies with patient demographics and extra-axial features. The extra-axial features of SpA include peripheral arthritis, uveitis, enthesitis, psoriasis, and inflammatory bowel disease (IBD) [3]. The prognosis and the response to treatment of axial SpA may differ according to the disease phenotype [4]. Previous studies have indicated that, in general, human leucocyte antigen (HLA)-B27 positivity, younger age, shorter disease duration, male sex, and elevated C-reactive protein (CRP) levels are associated with higher response rates to tumour necrosis factor alpha inhibitors (TNFi) in patients with axial SpA, whereas obesity and smoking are associated with lower response rates [5–8].

In view of the fact that the prognosis differs according to the disease phenotype, attempts have been made to classify patients into subgroups based on SpA features and demographic characteristics. As the relationships between clinical variables and phenotypes are not linear but rather complex, machine learning methods have become increasingly popular tools for classifying patients into subgroups [9–12]. Machine learning methods can be broadly divided into supervised learning, unsupervised learning, and reinforcement learning. Unsupervised learning is an appropriate method to use when performing classification without prior knowledge [13]. The representative unsupervised learning methods include hierarchical clustering and *k*-means clustering. These methods have been implemented to divide patients with SpA into subgroups using various clinical information. Previous studies have described the differences in disease activity [14, 15], radiographic progression [16], and functional assessment [17] between the subgroups classified according to distinctive features. Therefore, unsupervised learning is a useful tool for classifying patients with SpA.

The Korean College of Rheumatology Biologics and Targeted Therapy (KOBIO) registry is a prospective nationwide biologic therapy registry in South Korea. The KOBIO registry collects baseline data and follow-up data of patients using TNFi from multiple centres, thus allowing the analysis of a larger number of patients than previous studies. In this study, we performed an unsupervised learning analysis of patients included in the KOBIO registry using baseline characteristics. All patients in the registry were receiving TNFi, enabling the comparison of treatment responses and changes in disease activity between subgroup classifications.

## Methods

### Study population

The data for this study were retrieved from the KOBIO registry [18], a prospective nationwide biologic and targeted therapy registry for rheumatoid arthritis, axial SpA, and psoriatic arthritis with 34 participating hospitals in South Korea. This registry collects data on patients who started biologics or targeted synthetic disease-modifying anti-rheumatic drugs, including baseline clinical data and annual follow-up data. Our study cohort included patients with axial SpA who were enrolled in the registry between December 2012 and February 2019, who started TNFi for the first time (TNFi-naive patients), and who were followed up at least once after starting TNFi. All patients met the modified New York Criteria for AS or the Assessment of SpondyloArthritis International Society (ASAS) axial SpA criteria. Patients with insufficient baseline clinical data for cluster analysis were excluded.

### Clinical characteristics

The baseline variables selected for analysis were the most characteristic and frequent features of patients with SpA, such as those included in the ASAS classification criteria for axial SpA [19, 20]. We did not include sacroiliitis on magnetic resonance imaging (MRI) in the cluster analysis because 712 of the total of 1042 patients (68.3%) did not have MRI results. The following variables were selected for both multiple correspondence analysis (MCA) and cluster analysis: sex, age at disease onset (< 40 or ≥ 40 years), duration of disease symptoms (< 2 or ≥ 2 years), HLA-B27 positivity, inflammatory back pain, sacroiliitis detected on radiography according to the modified New York Criteria, peripheral arthritis, enthesitis, uveitis, psoriasis, IBD, and response to non-steroidal anti-inflammatory drugs (NSAIDs). More information is provided in the Supplementary Methods.

### Disease activity and treatment outcome

We did not include disease activity in clustering because it changes during the disease course, unlike the specific features of each patient. However, to more precisely describe each cluster, we also compared the disease activity of each cluster. The disease activity measures included levels of acute-phase reactants, such as erythrocyte sedimentation rate (ESR) and CRP; physical examination findings, such as swollen joint count (SJC) and tender joint count (TJC); and disease-related activity/severity composite scores, such as the Bath AS Disease Activity Index (BASDAI), Bath AS Functional Index (BASFI), AS Disease Activity Score based on ESR (ASDAS-ESR), and AS Disease Activity Score based on CRP (ASDAS-CRP). Furthermore, because all patients started TNFi at baseline, the treatment responses to TNFi at the first follow-up (1 year after starting TNFi) could be compared using the previously mentioned disease activity measures.

### Drug survival probabilities of TNFi

We compared the drug survival probabilities of TNFi between the clusters. The KOBIO registry collects information on current TNFi use and requires the participating hospitals to report the discontinuation of use. The registry contains up to 7 years of follow-up data. In the drug survival analysis of patients using TNFi, we included only cases of discontinuation owing to insufficient treatment response or adverse effects of the medications and excluded cases of discontinuation for other reasons, which mostly involved poor compliance and economic problems. However, these excluded cases were included in the sensitivity analysis comparing the drug survival probabilities of TNFi. In addition, we did not consider cases of remission to be TNFi discontinuation cases because these were the opposite of cases of discontinuation owing to inefficacy or adverse effects of treatment. We also performed a sensitivity analysis in which remission cases were considered as discontinuation cases.

### Comparison of the divided clusters with radiographic classification and grouping according to HLA-B27 positivity

We compared the divided clusters with well-known factors for dividing patients with axial SpA. First, we compared the divided clusters with the radiographic classifications of axial SpA. Axial SpA consists of radiographic axial SpA and non-radiographic axial SpA (i.e., does not meet the modified New York Criteria for sacroiliitis on radiography). In addition, non-radiographic axial SpA was divided into imaging and clinical arms according to the ASAS criteria for axial SpA. Second, we compared the divided clusters with grouping according to HLA-B27 positivity. Because previous studies have reported the differences in clinical features depending on radiographic changes and the presence or absence of HLA-B27, we investigated the correlation of the aforementioned variables with the divided clusters.

### Statistical analysis

#### Multiple correspondence analysis

MCA was used to graphically assess the associations of the analysed variables. MCA identifies composite dimensions in large categorical data sets in a manner analogous to how principal component analysis is used to identify latent variables in continuous data [21]. MCA is performed by applying the correspondence analysis algorithm to an indicator matrix [22]. An indicator matrix is an (individuals × variables) matrix in which rows represent individuals and columns represent categories of variables. Associations between variables are revealed by calculating the chi-square distance between the individuals and between the different categories of variables. Thereafter, these associations are graphically depicted to simplify the interpretation of the structures in the data. To uncover the underlying dimensions that best describe the central oppositions in the data, oppositions between rows and columns are maximized. The first axis is the most important dimension, the second axis is the second most important dimension, and so forth, in terms of the amount of variance accounted for. After clustering, MCA was used to assess whether the divided groups were well distinguished and how the variables affected each other before additional analysis on the divided groups was performed.

#### Classification methods

We used divisive hierarchical cluster analysis [23] to identify subgroups of patients with similar characteristics. Hierarchical clustering methods are categorized into agglomerative (bottom-up) and divisive (top-down) procedures. Divisive procedures begin by considering a group that includes all samples, which is divided into two groups in subsequent stages until all groups comprise only a single sample [24]. We used Euclidean distances to calculate the dissimilarities between observations. The optimum number of clusters was determined according to the average silhouette width [25, 26].

#### Comparison of clinical characteristics and drug survival probabilities between clusters

Differences in clinical characteristics, treatment responses, and TNFi discontinuation between clusters were tested using Student’s *t*-test for continuous variables and the chi-square test for categorical variables. The *p*-values of baseline clinical characteristics and treatment responses were adjusted using Holm–Bonferroni correction for multiple comparisons. Drug survival analysis was performed using the Kaplan–Meier method. All tests were two-sided, and *p* < 0.05 was considered to indicate statistical significance. All analyses were performed using R version 3.6.3 [27].

## Results

This study included 1042 patients with sufficient clinical data. The cohort showed a male predominance (76.1%), and the mean patient age was 38.32 (standard deviation [SD] 13.08) years. HLA-B27 positivity was observed in 89.2% of the patients. A total of 934 (89.6%) patients showed radiographic sacroiliitis that fulfilled the modified New York Criteria for AS. MRI was performed in 330 (31.7%) patients, and 255 (77.3%) patients showed sacroiliitis on MRI (Table S1). The most frequent extra-axial symptom was peripheral arthritis (37.2%), followed by uveitis (21.3%). At baseline, the mean BASDAI was 6.03 (SD 1.91), and the mean ESR and CRP level were 37.87 (SD 30.03) mm/h and 2.27 (SD 2.95) mg/dL, respectively (Table 1). The numbers of users of each TNFi were as follows: 425, 155, 260, and 202 patients were users of adalimumab, etanercept and its biosimilar agents, infliximab and its biosimilar agents, and golimumab, respectively (Table S2).

**Table 1:** Clinical characteristics of the cohort and comparison of characteristics according to clusters

	All patients (***n*** = 1042)	Axial group (***n*** = 828)	Extra-axial group (***n*** = 214)	***p***-value
Age at starting TNFi (years, %)	38.32 (13.08)	38.18 (12.76)	38.85 (14.28)	0.618
Sex (male, %)	793 (76.1)	651 (78.6)	142 (66.4)	0.001
Late onset (age ≥ 40 years, %)	329 (31.6)	247 (29.8)	82 (38.3)	0.034
Long disease duration (≥ 2 years, %)	426 (40.9)	380 (45.9)	46 (21.5)	< 0.001
HLA-B27 positivity (%)	929 (89.2)	740 (89.4)	189 (88.3)	0.782
Inflammatory back pain (%)	887 (85.1)	704 (85.0)	183 (85.5)	0.943
Radiographic sacroiliitis (%)	934 (89.6)	745 (90.0)	189 (88.3)	0.618
Peripheral arthritis (%)	388 (37.2)	203 (24.5)	185 (86.4)	< 0.001
Enthesitis (%)	218 (20.9)	68 (8.2)	150 (70.1)	< 0.001
Uveitis (%)	222 (21.3)	168 (20.3)	54 (25.2)	0.197
Psoriasis (%)	27 (2.6)	16 (1.9)	11 (5.1)	0.029
IBD (%)	13 (1.2)	9 (1.1)	4 (1.9)	0.618
Good response to NSAIDs (%)	358 (34.4)	212 (25.6)	146 (68.2)	< 0.001
csDMARD use (%)	107 (10.3)	75 (9.1)	32 (15.0)	0.029
NSAID use (%)	904 (86.8)	714 (86.2)	190 (88.8)	0.486
SJC	0.65 (2.28)	0.50 (2.37)	1.21 (1.80)	< 0.001
TJC	1.04 (3.06)	0.71 (2.67)	2.32 (4.00)	< 0.001
BASDAI	6.03 (1.91)	5.94 (1.92)	6.37 (1.81)	0.005
BASFI	3.47 (2.56)	3.42 (2.56)	3.66 (2.56)	0.305
ASDAS-ESR	3.74 (1.03)	3.65 (1.00)	4.05 (1.08)	< 0.001
ASDAS-CRP	3.66 (1.02)	3.61 (1.00)	3.87 (1.07)	0.003
ESR (mm/h)	37.87 (30.03)	35.65 (28.76)	46.46 (33.21)	< 0.001
CRP (mg/dL)	2.27 (2.95)	2.11 (2.74)	2.89 (3.60)	0.006

*TNFi* tumour necrosis factor alpha inhibitor, *HLA* human leucocyte antigen, *IBD* inflammatory bowel disease, *NSAID* non-steroidal anti-inflammatory drug, *csDMARD* conventional synthetic disease-modifying anti-rheumatic drug, *SJC* swollen joint count, *TJC* tender joint count, *BASDAI* Bath Ankylosing Spondylitis Disease Activity Index, *BASFI* Bath Ankylosing Spondylitis Functional Index, *ESR* erythrocyte sedimentation rate, *CRP* C-reactive protein, *ASDAS-ESR* Ankylosing Spondylitis Disease Activity Score based on ESR, *ASDAS-CRP* Ankylosing Spondylitis Disease Activity Score based on CRP

### Multiple correspondence analysis

**Fig. 1:**
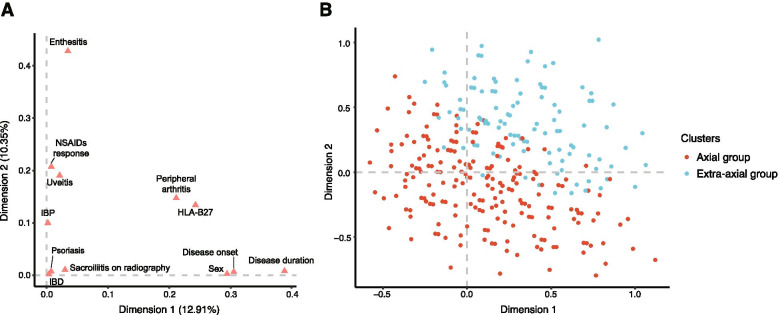
Multiple correspondence analysis (MCA) performed in all 1042 patients who met the inclusion criteria. First factorial plane with the *x*-axis and *y*-axis representing the first and the second most important dimensions, respectively. **A** Visualization of the correlation between variables and MCA principal dimensions. **B** Visualization of the distribution of individuals in the plane of the two major dimensions according to the divided clusters. HLA, human leucocyte antigen; IBD, inflammatory bowel disease; NSAIDs, non-steroidal anti-inflammatory drugs

MCA was used to graphically assess the patterns of patients according to the combination of clinical characteristics (Fig. 1). The correlations between the variables and principal dimensions are shown in Fig. 1A. The first dimension concerned items related to general conditions, which included disease duration, age at disease onset, and sex, whereas the second dimension seemed to be more related to the topography of the disease, including enthesitis, uveitis, and inflammatory back pain. Interestingly, although peripheral arthritis is a clinical characteristic of the disease and HLA-B27 positivity is a general condition, these factors are related to both dimensions. The coordinates of the variable categories are shown in Figure S1. The figure indicates a positive association among peripheral arthritis, psoriasis, and IBD. Remarkably, patients with uveitis were separated from the others, although this manifestation seemed to be most associated with enthesitis, which was also rather distinct from the other SpA features. In addition, HLA-B27-negative patients markedly differed from the other patients.

### Cluster analysis

Hierarchical clustering was performed to divide the entire cohort into subgroups. The analysis resulted in an optimal division of the sample into two clusters consisting of 828 and 214 patients, respectively. The variables that significantly differed between the two clusters were sex, disease onset after age 40 years, disease duration of > 2 years, peripheral arthritis, enthesitis, psoriasis, and good response to NSAIDs (Table 1). Group 1 (axial group) included many patients with a predominantly isolated axial disorder, male patients, and patients with a longer disease duration. Group 2 (extra-axial group) included patients showing a more diffuse pattern of disease with a high proportion of peripheral manifestations, including peripheral arthritis, enthesitis, and psoriasis, and a higher proportion of those with a good response to NSAIDs.

Interestingly, other variables not included in the cluster analysis were also differently distributed between the two clusters. Disease activity measures, including disease-related composite scores (BASDAI, ASDAS-ESR, and ASDAS-CRP), physical examination findings (SJC and TJC), and laboratory results (ESR and CRP level), were consistently high in the extra-axial group. However, BASFI, a composite score for measuring functionality, did not differ between the two groups. In addition, although the axial group consisted of patients with a predominantly axial disease, the two groups had a similar proportion of patients with inflammatory back pain and sacroiliitis on radiography.

### Disease prognosis in each cluster

We compared the disease prognosis between the two groups at 1-year follow-up (Table 2). No significant differences in disease activity or functional scores were observed between the two groups although the extra-axial group had higher disease activity scores at baseline. The differences in scores between baseline and follow-up were significantly different between the two groups in the case of SJC, TJC, ESR, CRP level, ASDAS-ESR, and ASDAS-CRP. In contrast, the differences in BASDAI, BASFI, and the proportion of patients who achieved 20% improvement according to the ASAS criteria (ASAS 20) were not significantly different between the two groups. Therefore, the main differences in disease prognosis were observed in terms of peripheral arthritis and laboratory results rather than the axial symptoms represented by BASDAI.

**Table 2:** Comparison of disease activity after 1-year follow-up and change of disease activity according to clusters

	Axial group (***n*** = 828)	Extra-axial group (***n*** = 214)	***p***-value
SJC at follow-up	0.12 (1.26)	0.23 (1.07)	0.221
TJC at follow-up	0.24 (1.63)	0.72 (3.46)	0.104
BASDAI at follow-up	2.60 (2.11)	2.63 (2.18)	0.840
BASFI at follow-up	1.53 (1.91)	1.32 (1.71)	0.187
ASDAS-ESR at follow-up	1.76 (1.00)	1.78 (0.95)	0.840
ASDAS-CRP at follow-up	1.61 (1.04)	1.48 (0.97)	0.123
ESR at follow-up (mm/h)	12.04 (15.49)	12.29 (14.99)	0.840
CRP at follow-up (mg/dL)	0.46 (0.98)	0.42 (1.05)	0.704
Δ SJC	− 0.37 (1.78)	− 0.98 (1.98)	< 0.001
Δ TJC	− 0.46 (2.08)	− 1.60 (2.27)	< 0.001
Δ BASDAI	− 3.36 (2.50)	− 3.75 (2.64)	0.104
Δ BASFI	− 1.92 (2.37)	− 2.34 (2.63)	0.096
Δ ASDAS-ESR	− 1.90 (1.25)	− 2.27 (1.34)	0.001
Δ ASDAS-CRP	− 2.00 (1.28)	− 2.40 (1.43)	0.001
Δ ESR (mm/h)	− 23.65 (28.45)	− 34.17 (31.38)	< 0.001
Δ CRP (mg/dL)	− 1.62 (2.72)	− 2.41 (3.62)	0.014
ASAS 20	444 (60.6)	131 (66.5)	0.196

*SJC* swollen joint count, *TJC* tender joint count, *BASDAI* Bath Ankylosing Spondylitis Disease Activity Index, *BASFI* Bath Ankylosing Spondylitis Functional Index, *ESR* erythrocyte sedimentation rate, *CRP* C-reactive protein, *ASDAS-ESR* Ankylosing Spondylitis Disease Activity Score based on ESR, *ASDAS-CRP* Ankylosing Spondylitis Disease Activity Score based on CRP, *ASAS 20* Assessment of SpondyloArthritis International Society 20% improvement criteria

### Drug survival probabilities of TNFi in each group

**Fig. 2:**
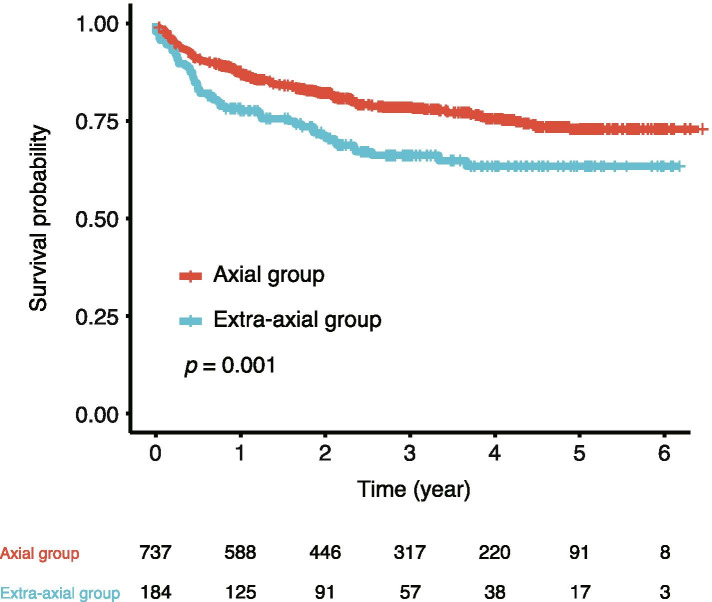
Drug survival probabilities of tumour necrosis factor alpha inhibitors. Kaplan–Meier survival analysis was used for comparisons between the axial and extra-axial groups

We compared the drug survival probabilities between the two groups. We excluded patients who stopped TNFi for reasons other than inefficacy or adverse events, because most of such patients stopped TNFi owing to economic problems and low compliance. The number of patients included in the drug survival analysis was 737 and 184 in the axial and extra-axial groups, respectively. The mean duration of follow-up was 2.67 (SD 1.73) years. The axial group had a significantly higher drug survival probability of TNFi than the extra-axial group in the Kaplan–Meier survival analysis (*p* = 0.001, Fig. 2). We compared the reasons for the discontinuation of TNFi and the duration until discontinuation between the two groups (Table 3). The number of patients with treatment inefficacy was significantly greater in the extra-axial group (11.8% vs. 19.0%, *p* = 0.014). The duration until discontinuation in patients with adverse events was significantly shorter in the extra-axial group (0.97 vs. 0.61 years, *p* = 0.049). The duration until discontinuation in patients with treatment inefficacy and the number of patients with adverse events were not significantly different between the two clusters; however, they showed similar trends.

**Table 3:** The reason for discontinuation of the tumour necrosis factor alpha inhibitors and the duration until discontinuation of the two groups that were included in the drug survival analysis

		All patients (***n*** = 921)	Axial group (***n*** = 737)	Extra-axial group (***n*** = 184)	***p***-value
Inefficacy	*n* (%)	122 (13.2)	87 (11.8)	35 (19.0)	0.014
	Duration (years)	1.22 (1.20)	1.30 (1.28)	1.01 (0.97)	0.181
Adverse events	*n* (%)	95 (10.3)	72 (9.8)	23 (12.5)	0.337
	Duration (years)	0.89 (0.92)	0.97 (0.98)	0.61 (0.67)	0.049

For sensitivity analysis, we compared the drug survival probabilities between the two groups without excluding patients who stopped TNFi for reasons other than inefficacy or adverse events. In this analysis, all patients had drug survival information, and the mean duration of follow-up was 2.53 (SD 1.72) years. The axial group still had a significantly higher drug survival probability (*p* < 0.001, Figure S2A) in the sensitivity analysis. In addition, we counted patients who stopped TNFi treatment because of remission as discontinuation cases. The axial group had a significantly higher drug survival probability (*p* = 0.001, Figure S2B). The number of patients who discontinued TNFi because of remission and other reasons is shown in Table S3.

### Comparison of the divided clusters with radiographic classification and grouping according to HLA-B27 positivity

We compared the divided clusters with well-known factors for dividing patients with axial SpA. First, we divided the patients with axial SpA according to radiographic classifications: radiographic and non-radiographic axial SpA (Table S4). Patients with non-radiographic axial SpA were further divided into the imaging and clinical arms according to the ASAS criteria for axial SpA (Table S5). The axial and extra-axial groups were not distributed along with the radiographic classifications. The drug survival probability of TNFi did not differ between radiographic and non-radiographic axial SpA (Figure S3A). Second, we divided the patients with axial SpA according to HLA-B27 positivity (Table S6). Similarly, the axial and extra-axial groups were not distributed along with HLA-B27 positivity. However, HLA-B27 positivity had a little correlation with peripheral features, whereas the baseline demographics differed according to HLA-B27 positivity. In addition, the drug survival probability of TNFi was significantly different according to HLA-B27 positivity (Figure S3B).

## Discussion

In this study, cluster analysis of patients with axial SpA treated with TNFi identified two groups with distinctive characteristics: one group was characterized by a predominantly isolated axial disorder with earlier disease onset and longer disease duration, and the other was characterized by more frequent peripheral SpA features. Furthermore, the two groups had different disease activity at baseline, treatment responses, and drug survival probabilities of TNFi, which were not included as input variables for classification. To the best of our knowledge, this is the first cluster analysis performed in patients with axial SpA or related conditions that includes a comparison of treatment responses and a drug survival analysis between subgroup classifications.

Axial SpA is clinically known to be a heterogeneous group of related disorders with similar clinical, genetic, pathological, and likely etiological features [28]. The group of conditions includes AS, psoriatic arthritis, reactive arthritis, and IBD-associated arthritis, according to the most prominent features of patients. However, these categories are not expected to reflect the underlying pathogenesis. Therefore, several attempts have been made to classify SpA according to the combination of symptoms [14, 15].

The first step in our analysis, MCA, revealed two main axes that maximize the variance of individuals. Of the two principal dimensions, one correlated with general conditions and the other with the characteristic disease phenotype. However, peripheral arthritis and HLA-B27 positivity were related to both dimensions. The coordinates of variable categories showed that peripheral arthritis, psoriasis, and IBD were grouped together, whereas uveitis was separated from the rest of the SpA features.

Cluster analyses using disease manifestations have been performed in two previous studies in patients with overall SpA [14] or early inflammatory back pain suggestive of SpA [15]. Both studies identified two groups characterized by different distributions of peripheral manifestations: one with a predominantly isolated axial disorder and the other with a more diffuse pattern of disease, consistent with the present study. However, uveitis did not differ between the two groups in all studies. Because other peripheral manifestations such as peripheral arthritis, enthesitis, and psoriasis were consistently well clustered in all studies, uveitis was considered to be differently distributed from other peripheral manifestations. In addition, there were more patients with a late disease onset in the extra-axial group in this study. The above-mentioned previous studies reported inconsistent results in terms of disease onset. The first study showed more patients with late onset in the axial group, in contrast with the present study, whereas the second study showed similar results to the present study. Finally, axial involvement (buttock pain in both previous studies and radiographic sacroiliitis in the first study) was not different between the two groups in all studies, including ours. The presence of axial involvement may not affect the classification of patients with SpA. However, it should be considered that the present study only targeted patients with axial SpA, and about 90% of the patients had inflammatory back pain and radiographic sacroiliitis. Therefore, clustering of axial symptoms may not be appropriate based on this study. Overall, the characteristics of clusters divided according to disease phenotypes were mainly consistent with those in previous studies, although the participants of each study were somewhat different.

This study did not include information that reveals the background of this clustering. However, previous studies have conducted similar cluster analyses on SpA manifestations in familial SpA [14, 29]. They observed a trend of familial aggregation by clusters. Accordingly, previous studies have suggested that the clustering of clinical features may be caused by genetic factors. We obtained clustering results that were similar to those of previous studies. Therefore, the clustering in our study may also be based on genetic background.

The extra-axial group had higher disease activity at baseline in terms of composite disease activity scores, physical examination of joints, and acute-phase reactant levels. Previous studies have reported higher disease activity in patients with a higher frequency of peripheral manifestations [30–32]. In addition, previous cluster analysis showed higher disease activity scores and more frequently elevated acute-phase reactant levels in the group with more peripheral manifestations [15]. Although disease activity was not included in the input variables for the cluster analysis in this study, patients in the extra-axial group had higher disease activity, consistent with previous studies. After 1 year of treatment with TNFi, the difference in disease activity between the two groups disappeared, and patients in both groups similarly achieved ASAS 20. Therefore, the 1-year response to TNFi was comparable between the two groups regardless of disease manifestations and disease activity. However, drug survival analysis revealed that patients in the extra-axial group had significantly more withdrawals of TNFi than the others in the long-term follow-up. Some explanations for these findings can be proposed. First, the drug survival analysis included information of patients who discontinued TNFi after 1 year or more but were not included in the analysis of disease activity at the 1-year follow-up. Second, although the difference in the number of adverse events between the two groups did not reach statistical significance, the greater number of adverse events in the extra-axial group could have affected the results to some extent. The occurrence of adverse events could explain why the treatment was not maintained despite comparable disease activities. Third, the time of discontinuation was earlier in the extra-axial group in both patients with inefficacy and those with adverse events. In the Kaplan–Meier analysis, not only the number of events but also the time of discontinuation is an important factor. Fourth, non-inflammatory joint symptoms could have contributed to the lower treatment response in the extra-axial group. The KOBIO registry defined the presence of peripheral arthritis based on the findings of physical examination by clinicians, in accordance with most large-scale clinical studies. However, it is occasionally difficult to discriminate between inflammatory and non-inflammatory arthritis through physical examination alone. Because non-inflammatory joint symptoms do not respond to TNFi, this could have influenced the lower drug survival rates in the extra-axial group despite the comparable disease activities at 1 year after treatment. These factors can be considered the reasons for the difference in the drug survival probabilities of TNFi in the Kaplan–Meier analysis.

We compared the divided clusters with radiographic classification and grouping according to HLA-B27 positivity. Various demographic and clinical differences have been reported between patients with radiographic and non-radiographic axial SpA [33, 34] and between HLA-B27-positive and HLA-B27-negative patients [35]. We divided our cohort according to radiographic classifications and HLA-B27 positivity and compared these divisions with the two clusters. We found that the axial and extra-axial groups were not distributed according to radiographic classifications or HLA-B27 positivity. Therefore, the divided clusters cannot be explained by these factors. In addition, the presence of HLA-B27 affected several patient demographics and the drug survival probability of TNFi. Although HLA-B27 positivity differed from other SpA features, as shown in the MCA, it can be an important factor when classifying patients with axial SpA.

Disease duration can affect the clinical presentation of SpA, with increased disease duration leading to increased frequencies of peripheral manifestations [29, 36]. Thus, disease duration may be a confounding factor in the clustering of peripheral manifestations. However, patients with a long disease duration (> 2 years) were in significantly higher proportion in the axial group. Therefore, it is unlikely that disease duration influenced the clustering of peripheral manifestations in this study.

The strengths of our study include its multi-centre design and the large number of analysed patients. Furthermore, because all patients started TNFi at baseline and the study had a prospective design, we could analyse the differences in treatment response and survival, which was not possible in previous cluster analysis studies. Nevertheless, this could also be a limitation of this study because the study population included only patients with an active disease and thus may not be representative of the general axial SpA population. In addition, we could not consider the use of glucocorticoids because the KOBIO registry did not contain information about it. However, because systemic glucocorticoids are rarely used in patients with axial SpA in South Korea, according to most guidelines, we expect that the effect of glucocorticoids to be minimal.

## Conclusion

In conclusion, cluster analysis of patients with axial SpA identified at baseline showed two different clinical phenotypes: one with predominantly axial manifestations and the other with more frequent peripheral manifestations and higher disease activity. The results of the present study were generally consistent with those of previously performed cluster analyses. After 1 year of treatment with TNFi, the treatment response was similar between the two groups and the disease activity was comparable between the two clusters. However, the long-term drug survival probabilities of TNFi were significantly lower in the extra-axial group than in the axial group.

## Supplementary Information

**Additional file 1: **Supplementary Methods. **Table S1**: Clinical variables excluded in adjusting multiple comparisons. **Table S2**: Number of users of each TNFi. **Table S3**: Reasons for discontinuation of tumour necrosis factor alpha inhibitors and duration until discontinuation in two groups that were excluded from the drug survival analysis. **Table S4**: Comparison of the divided clusters with radiographic classification (radiographic axial SpA and non-radiographic axial SpA). **Table S5**: Comparison of the divided clusters with the division of non-radiographic axial SpA into radiographic and clinical arms according to the Assessment of SpondyloArthritis International Society criteria for axial SpA. **Table S6**: Comparison of the divided clusters with grouping according to HLA-B27 positivity.

**Additional file 2: Figure S1**: Results of multiple correspondence analysis (MCA). The first factorial plane with the x-axis and y-axis represents the first and second most important dimensions, respectively. Visualisation of the coordinates of each variable category. Each symbol represents the presence or absence of the variables. **Figure S2**: Results of sensitivity analyses. Drug survival probabilities of tumour necrosis factor alpha inhibitors (TNFi) in each group: (A) including patients who stopped TNFi for reasons other than inefficacy or adverse event; (B) including patients who stopped TNFi owing to remission. **Figure S3**: Drug survival probability of tumour necrosis factor alpha inhibitors (TNFi) according to (A) radiographic classification and (B) human leukocyte antigen (HLA)-B27 positivity. **Figure S4**: Results of factor analysis of mixed data (FAMD) at the individual level divided by cluster analysis. In this cluster analysis, age at disease onset and duration of disease symptoms were used as continuous values. **Figure S5**: Graph of average silhouette width according to the number of clusters.

## Data Availability

The data that support the findings of this study are available from the Korean College of Rheumatology, but restrictions apply to the availability of these data, which were used under licence for the current study, and so are not publicly available. Data are however available from the authors upon reasonable request and with permission from the Korean College of Rheumatology.

## Abbreviations

SpA: Axial spondyloarthritis
AS: Ankylosing spondylitis
IBD: Inflammatory bowel disease
HLA: human leucocyte antigen
CRP: C-reactive protein
TNFi: Tumour necrosis factor alpha inhibitors
KOBIO: Korean College of Rheumatology Biologics and Targeted Therapy
ASAS: Assessment of SpondyloArthritis International Society
ESR: Erythrocyte sedimentation rate
SJC: Swollen joint count
TJC: Tender joint count
BASDAI: Bath AS Disease Activity Index
BASFI: Bath AS Functional Index
ASDAS-ESR: AS Disease Activity Score based on ESR
ASDAS-CRP: AS Disease Activity Score based on CRP
