# Supplementary material for: A cluster analysis of patients with axial spondyloarthritis using tumour necrosis factor alpha inhibitors based on clinical characteristics
Source: Arthritis Res Ther. 2021 Nov 15;23:284. doi: 10.1186/s13075-021-02647-z (PMC8591959; doi:10.1186/s13075-021-02647-z)
Supplement: Supplementary file 1 — Additional file 1: Supplementary Methods. Table S1: Clinical variables excluded in adjusting multiple comparisons. Table S2: Number of users of each TNFi. Table S3: Reasons for discontinuation of tumour necrosis factor alpha inhibitors and duration until discontinuation in two groups that were excluded from the drug survival analysis. Table S4: Comparison of the divided clusters with radiographic classification (radiographic axial SpA and non-radiographic axial SpA). Table S5: Comparison of the divided clusters with the division of non-radiographic axial SpA into radiographic and clinical arms according to the Assessment of SpondyloArthritis International Society criteria for axial SpA. Table S6: Comparison of the divided clusters with grouping according to HLA-B27 positivity. [file 13075_2021_2647_MOESM1_ESM.docx]

**Supplementary material**

**A cluster analysis of patients with axial spondyloarthritis using tumour necrosis factor alpha inhibitors based on clinical characteristics**

**Seulkee Lee^1^, Seonyoung Kang^1^, Yeonghee Eun^1^, Hong-Hee Won^2^, Hyungjin Kim^1^, Hoon-Suk Cha^1^, Eun-Mi Koh^1^, Jaejoon Lee^1^**

^1^Department of Medicine, Samsung Medical Center, Sungkyunkwan University School of Medicine, Seoul, Republic of Korea

^2^Samsung Advanced Institute for Health Sciences & Technology (SAIHST), Sungkyunkwan University, Samsung Medical Center, Seoul, Republic of Korea

**Supplementary Methods**

**Clinical characteristics: variable selection**

In a previous study by Costantino *et al.* [1], age at disease onset and duration of disease symptoms were converted into a binary format. However, because these two factors are continuous variables, hierarchical clustering analysis is possible without converting them into binary variables. We assessed the results of the cluster analysis to determine whether the binary forms were appropriate for these variables. Continuous variables have different scales compared with binary variables. Before performing the cluster analysis, the continuous variables were normalized to match the scale of the binary variables. The divided clusters were analysed through factor analysis of mixed data (FAMD) rather than multiple correspondence analysis (MCA) because MCA is applicable only when all variables are binary. Given the results of FAMD, the clusters did not appear to be clearly separated (Figure S2). For this reason, we decided to convert age at disease onset and duration of disease symptoms into a binary format, following the previous study.

**Statistical analysis: hierarchical cluster analysis**

We used the ‘cluster’ package [2] in R for divisive hierarchical clustering analysis, specifically the DIANA (DIvisive ANAlysis) function. The input distance matrix between observations was calculated using the ‘dist’ function in R, which is included in the ‘stats’ package [3]. The DIANA function does not have options for the linkage method. In the DIANA function, clusters are divided based on the maximum average dissimilarity, which is highly similar to the mean or average linkage clustering method [4].

The optimum number of clusters was determined according to the average silhouette width (ASW). ASW was calculated using the ‘WeightedCluster’ package (version 1.4-1) in R [5]. ASW is based on the coherence of the assignment of an observation to a given group, comparing the average weighted distance of an observation from the other members of its group and its average weighted distance from the closest group. A value is calculated for each observation; however, more attention is paid to the average silhouette. A higher value of ASW is interpreted as a stronger structure. We calculated the ASW by varying the number of clusters (Figure S3). The ASW value was the highest at the number of clusters of two.

**Table S1** Clinical variables excluded in adjusting multiple comparisons

|  | | **All patients (*n* = 1042)** | **Cluster A**  **(*n* = 828)** | **Cluster B**  **(*n* = 214)** |
| --- | --- | --- | --- | --- |
| Disease duration (years) | | 3.61 (5.12) | 4.02 (5.34) | 2.00 (3.77) |
| Disease onset (years) | | 34.71 (13.31) | 34.16 (13.03) | 36.85 (14.19) |
| MRI examination (%) | | 330 (31.7) | 259 (31.3) | 71 (33.1) |
| Sacroiliitis on MRI (%) | | 255 (77.3) | 198 (76.4) | 57 (80.3) |
| Clinical criteria | Radiographic axial SpA (%) | 934 (89.6) | 745 (90.0) | 189 (88.3) |
|  | Non-radiographic axial SpA (%) | 108 (10.4) | 83 (10.0) | 25 (11.7) |
|  | Imaging arm* (%) | 52 (5.0) | 41 (5.0) | 11 (5.1) |
|  | Clinical arm* (%) | 56 (5.3) | 42 (5.1) | 14 (6.5) |
| *Imaging arm: sacroiliitis on imaging plus ≥ 1 SpA features; clinical arm: HLA-B27 positive plus ≥ 2 SpA features.  *MRI* magnetic resonance imaging; *SpA* spondyloarthritis. | | | | |

**Table S2** Number of users of each tumour necrosis factor alpha inhibitors.

|  | **All patients (*n* = 1042)** | **Cluster A (*n* = 828)** | **Cluster B (*n* = 214)** |
| --- | --- | --- | --- |
| Adalimumab | 425 (40.8) | 343 (41.4) | 82 (38.3) |
| Etanercept | 155 (14.9) | 123 (14.9) | 32 (15.0) |
| Infliximab | 260 (24.9) | 204 (24.6) | 56 (26.1) |
| Golimumab | 202 (19.4) | 158 (19.1) | 44 (20.6) |

**Table S3** Reasons for discontinuation of tumour necrosis factor alpha inhibitors and duration until discontinuation in two groups that were excluded from the drug survival analysis.

|  |  | **All patients**  **(*n* = 1042)** | **Axial group**  **(*n* = 828)** | **Extra-axial group (*n* = 214)** | ***P*-value** |
| --- | --- | --- | --- | --- | --- |
| Remission | *n* | 46 (4.4) | 36 (4.3) | 10 (4.7) | 0.984 |
|  | Duration (years) | 2.20 (1.45) | 2.13 (1.44) | 2.44 (1.51) | 0.568 |
| Other reasons | *n* | 120 (11.5) | 90 (10.9) | 30 (14.0) | 0.244 |
|  | Duration (years) | 1.46 (1.19) | 1.32 (1.06) | 1.88 (1.46) | 0.057 |

**Table S4** Comparison of the divided clusters with radiographic classification (radiographic axial SpA and non-radiographic axial SpA)

|  | **Radiographic axial SpA (*n* = 934)** | **Non-radiographic axial SpA (*n* = 108)** | ***P*-value** |
| --- | --- | --- | --- |
| Extra-axial group* (%) | 189 (20.2) | 25 (23.1) | 0.909 |
| Age at starting TNFi (years, %) | 38.80 (13.06) | 34.14 (12.58) | 0.003 |
| Sex (male, %) | 716 (76.7) | 77 (71.3) | 0.489 |
| Late onset (age ≥ 40 years, %) | 301 (32.2) | 28 (25.9) | 0.442 |
| Long disease duration (≥ 2 years, %) | 397 (42.5) | 29 (26.9) | 0.013 |
| HLA-B27 positivity (%) | 833 (89.2) | 96 (88.9) | > 0.999 |
| Inflammatory back pain (%) | 803 (86.0) | 84 (77.8) | 0.110 |
| Radiographic sacroiliitis (%) | 934 (100.0) | 0 (0.0) | NA |
| Peripheral arthritis (%) | 341 (36.5) | 47 (43.5) | 0.404 |
| Enthesitis (%) | 194 (20.8) | 24 (22.2) | > 0.999 |
| Uveitis (%) | 207 (22.2) | 15 (13.9) | 0.180 |
| Psoriasis (%) | 25 (2.7) | 2 (1.9) | > 0.999 |
| IBD (%) | 11 (1.2) | 2 (1.9) | > 0.999 |
| Good response to NSAIDs (%) | 319 (34.2) | 39 (36.1) | > 0.999 |
| csDMARD use (%) | 94 (10.1) | 13 (12.0) | 0.974 |
| NSAID use (%) | 805 (86.2) | 99 (91.7) | 0.354 |
| SJC | 0.64 (2.36) | 0.69 (1.50) | > 0.999 |
| TJC | 1.04 (3.14) | 1.06 (2.23) | > 0.999 |
| BASDAI | 6.04 (1.90) | 5.92 (1.92) | 0.893 |
| BASFI | 3.47 (2.58) | 3.49 (2.42) | > 0.999 |
| ASDAS-ESR | 3.77 (1.03) | 3.42 (0.99) | 0.004 |
| ASDAS-CRP | 3.69 (1.01) | 3.42 (1.06) | 0.053 |
| ESR (mm/h) | 39.08 (30.16) | 27.44 (26.79) | 0.001 |
| CRP (mg/dL) | 2.32 (2.98) | 1.86 (2.70) | 0.274 |

*According to the divided clusters in this study.

*SpA* spondyloarthritis; *TNFi* tumour necrosis factor alpha inhibitor, *HLA* human leucocyte antigen, *NA* not applicable, *IBD* inflammatory bowel disease, *NSAID* non-steroidal anti-inflammatory drug, *csDMARD* conventional synthetic disease-modifying anti-rheumatic drug, *SJC* swollen joint count, *TJC* tender joint count, *BASDAI* Bath Ankylosing Spondylitis Disease Activity Index, *BASFI* Bath Ankylosing Spondylitis Functional Index, *ESR* erythrocyte sedimentation rate, *CRP* C-reactive protein, *ASDAS-ESR* Ankylosing Spondylitis Disease Activity Score based on ESR, *ASDAS-CRP* Ankylosing Spondylitis Disease Activity Score based on CRP

**Table S5** Comparison of the divided clusters with the division of non-radiographic axial SpA into radiographic and clinical arms according to the Assessment of SpondyloArthritis International Society criteria for axial SpA

|  | **All patients**  **with nr-axSpA (*n* = 108)** | **Radiographic**  **arm**  **(*n* = 52)** | **Clinical arm**  **(*n* = 56)** | ***P*-value** |
| --- | --- | --- | --- | --- |
| Extra-axial group* (%) | 25 (23.1) | 11 (21.2) | 14 (25.0) | > 0.999 |
| Age at starting TNFi (years, %) | 34.14 (12.58) | 33.30 (13.11) | 34.92 (12.13) | 0.850 |
| Sex (male, %) | 77 (71.3) | 39 (75.0) | 38 (67.9) | 0.850 |
| Late onset (age ≥ 40 years, %) | 28 (25.9) | 15 (28.8) | 13 (23.2) | 0.873 |
| Long disease duration (≥ 2 years, %) | 29 (26.9) | 11 (21.2) | 18 (32.1) | 0.783 |
| HLA-B27 positivity (%) | 96 (88.9) | 40 (76.9) | 56 (100.0) | 0.011 |
| Inflammatory back pain (%) | 84 (77.8) | 47 (90.4) | 37 (66.1) | 0.060 |
| Peripheral arthritis (%) | 47 (43.5) | 25 (48.1) | 22 (39.3) | 0.850 |
| Enthesitis (%) | 24 (22.2) | 10 (19.2) | 14 (25.0) | 0.873 |
| Uveitis (%) | 15 (13.9) | 8 (15.4) | 7 (12.5) | 0.957 |
| Psoriasis (%) | 2 (1.9) | 2 (3.8) | 0 (0.0) | 0.850 |
| IBD (%) | 2 (1.9) | 1 (1.9) | 1 (1.8) | > 0.999 |
| Good response to NSAIDs (%) | 39 (36.1) | 23 (44.2) | 16 (28.6) | 0.739 |
| csDMARD use (%) | 13 (12.0) | 9 (17.3) | 4 (7.1) | 0.739 |
| NSAID use (%) | 99 (91.7) | 48 (92.3) | 51 (91.1) | > 0.999 |
| SJC | 0.69 (1.50) | 0.73 (1.76) | 0.64 (1.21) | 0.957 |
| TJC | 1.06 (2.23) | 1.19 (1.98) | 0.95 (2.45) | 0.850 |
| BASDAI | 5.92 (1.92) | 6.06 (1.95) | 5.78 (1.90) | 0.850 |
| BASFI | 3.49 (2.42) | 3.45 (2.35) | 3.53 (2.50) | 0.957 |
| ASDAS-ESR | 3.42 (0.99) | 3.52 (1.04) | 3.32 (0.94) | 0.783 |
| ASDAS-CRP | 3.42 (1.06) | 3.58 (1.03) | 3.27 (1.07) | 0.739 |
| ESR (mm/h) | 27.44 (26.79) | 29.48 (27.61) | 25.55 (26.11) | 0.850 |
| CRP (mg/dL) | 1.86 (2.70) | 2.23 (3.18) | 1.52 (2.14) | 0.739 |

*According to the divided clusters in this study.

*SpA* spondyloarthritis; nr-axSpA, non-radiographic axial SpA; *TNFi* tumour necrosis factor alpha inhibitor, *HLA* human leucocyte antigen, *IBD* inflammatory bowel disease, *NSAID* non-steroidal anti-inflammatory drug, *csDMARD* conventional synthetic disease-modifying anti-rheumatic drug, *SJC* swollen joint count, *TJC* tender joint count, *BASDAI* Bath Ankylosing Spondylitis Disease Activity Index, *BASFI* Bath Ankylosing Spondylitis Functional Index, *ESR* erythrocyte sedimentation rate, *CRP* C-reactive protein, *ASDAS-ESR* Ankylosing Spondylitis Disease Activity Score based on ESR, *ASDAS-CRP* Ankylosing Spondylitis Disease Activity Score based on CRP

**Table S6** Comparison of the divided clusters with grouping according to HLA-B27 positivity

|  | **HLA-B27 (+)**  **(*n* = 934)** | **HLA-B27 (-)**  **(*n* = 108)** | ***P*-value** |
| --- | --- | --- | --- |
| Extra-axial group* (%) | 189 (20.2) | 25 (23.1) | 0.812 |
| Age at starting TNFi (years, %) | 37.76 (12.76) | 42.87 (14.76) | 0.002 |
| Sex (male, %) | 725 (78.0) | 68 (60.2) | < 0.001 |
| Late onset (age ≥ 40 years, %) | 270 (29.1) | 59 (52.2) | < 0.001 |
| Long disease duration (≥ 2 years, %) | 398 (42.8) | 28 (24.8) | 0.001 |
| HLA-B27 positivity (%) | 929 (100.0) | 0 (0.0) | NA |
| Inflammatory back pain (%) | 795 (85.6) | 92 (81.4) | 0.162 |
| Radiographic sacroiliitis (%) | 833 (89.7) | 101 (89.4) | > 0.999 |
| Peripheral arthritis (%) | 338 (36.4) | 50 (44.2) | 0.252 |
| Enthesitis (%) | 198 (21.3) | 20 (17.7) | 0.638 |
| Uveitis (%) | 215 (23.1) | 7 (6.2) | < 0.001 |
| Psoriasis (%) | 22 (2.4) | 5 (4.4) | 0.501 |
| IBD (%) | 10 (1.1) | 3 (2.7) | 0.501 |
| Good response to NSAIDs (%) | 323 (34.8) | 35 (31.0) | 0.665 |
| csDMARD use (%) | 93 (10.0) | 14 (12.4) | 0.688 |
| NSAID use (%) | 804 (86.5) | 100 (88.5) | 0.788 |
| SJC | 0.62 (2.31) | 0.86 (2.05) | 0.467 |
| TJC | 0.93 (2.64) | 1.91 (5.31) | 0.133 |
| BASDAI | 6.02 (1.88) | 6.14 (2.08) | 0.688 |
| BASFI | 3.48 (2.56) | 3.40 (2.55) | 0.812 |
| ASDAS-ESR | 3.77 (1.04) | 3.48 (0.95) | 0.009 |
| ASDAS-CRP | 3.69 (1.03) | 3.40 (0.94) | 0.007 |
| ESR (mm/h) | 39.13 (30.25) | 27.52 (26.00) | < 0.001 |
| CRP (mg/dL) | 2.37 (3.03) | 1.40 (1.97) | < 0.001 |

*According to the divided clusters in this study.

*TNFi* tumour necrosis factor alpha inhibitor, *HLA* human leucocyte antigen, *NA* not applicable, *IBD* inflammatory bowel disease, *NSAID* non-steroidal anti-inflammatory drug, *csDMARD* conventional synthetic disease-modifying anti-rheumatic drug, *SJC* swollen joint count, *TJC* tender joint count, *BASDAI* Bath Ankylosing Spondylitis Disease Activity Index, *BASFI* Bath Ankylosing Spondylitis Functional Index, *ESR* erythrocyte sedimentation rate, *CRP* C-reactive protein, *ASDAS-ESR* Ankylosing Spondylitis Disease Activity Score based on ESR, *ASDAS-CRP* Ankylosing Spondylitis Disease Activity Score based on CRP

**References**

1. Costantino F, Aegerter P, Dougados M, Breban M, D'Agostino MA. Two Phenotypes Are Identified by Cluster Analysis in Early Inflammatory Back Pain Suggestive of Spondyloarthritis: Results From the DESIR Cohort. Arthritis Rheumatol. 2016;68(7):1660-8.

2. Maechler M, Rousseeuw P, Struyf A, Hubert M, Hornik K. Cluster: cluster analysis basics and extensions. R package version. 2012;1(2):56.

3. Team RC. R: A language and environment for statistical computing. 2013.

4. Kaufman L, Rousseeuw PJ. Finding groups in data: an introduction to cluster analysis. John Wiley & Sons; 2009.

5. Studer M. WeightedCluster library manual: A practical guide to creating typologies of trajectories in the social sciences with R. 2013.
