# Supplementary material for: A cluster analysis of patients with axial spondyloarthritis using tumour necrosis factor alpha inhibitors based on clinical characteristics
Source: Arthritis Res Ther. 2021 Nov 15;23:284. doi: 10.1186/s13075-021-02647-z (PMC8591959; doi:10.1186/s13075-021-02647-z)

Figure S1. Results of multiple correspondence analysis (MCA). The first factorial plane with the x-axis and y-axis represents the first and second most important dimensions, respectively. Visualisation of the coordinates of each variable category. Each symbol represents the presence or absence of the variables.

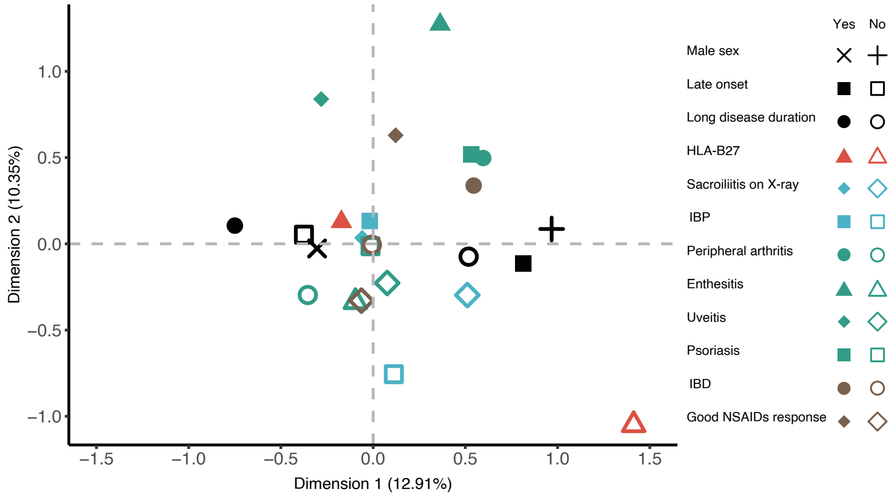

Figure S2. Results of sensitivity analyses. Drug survival probabilities of tumour necrosis inhibitors (TNFi) in each group: (A) including the patients who stopped TNFi for reasons other than inefficacy or adverse event; (B) including patients who stopped TNFi owing to remission.

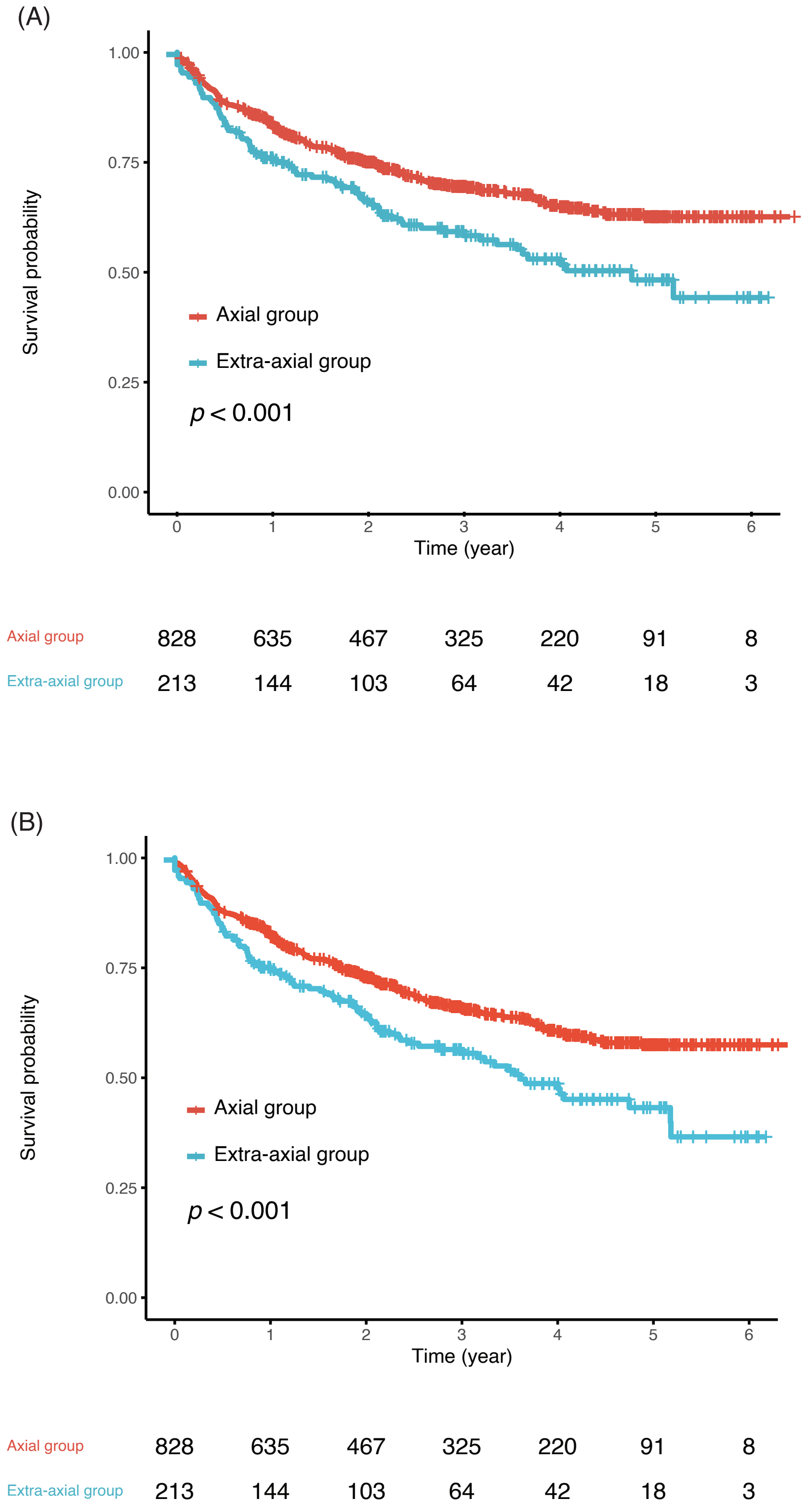

Figure S3. Drug survival probability of tumour necrosis factor alpha inhibitors (TNFi) according to (A) radiographic classification and (B) human leukocyte antigen (HLA)-B27 positivity.

(A)

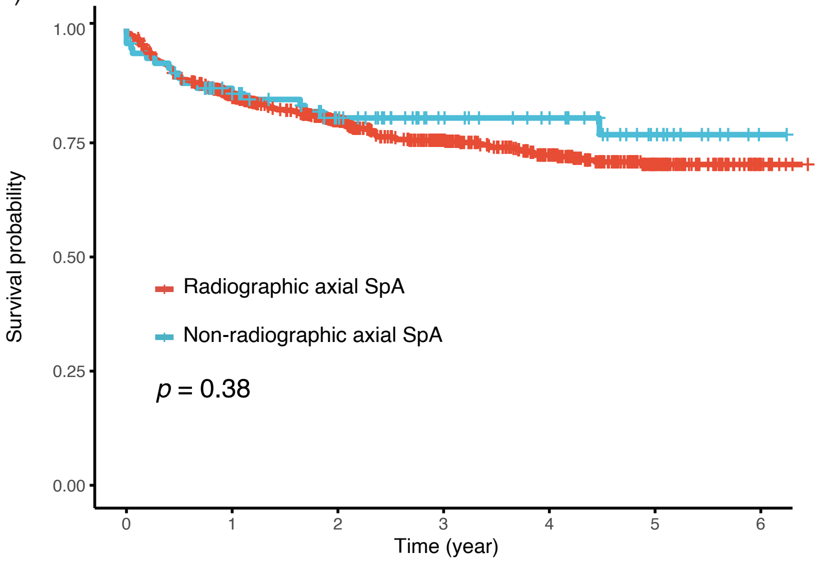

Radiographic axial SpA group	828	642	482	337	229	94	10
Non-radiographic axial SpA group	93	71	55	37	29	14	1

(B)

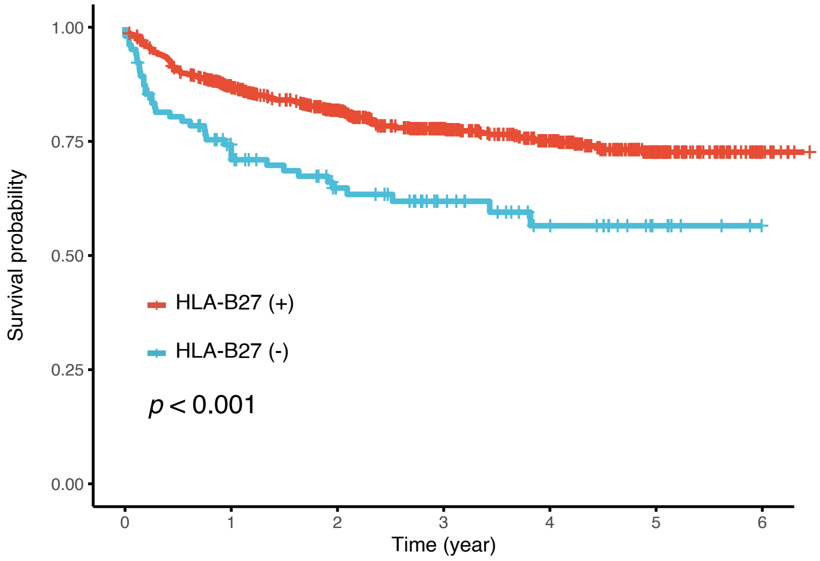

HLA-B27 positive group	818	649	491	344	240	101	11
HLA-B27 negative group	103	64	46	30	18	7	0

Figure S4. Results of factor analysis of mixed data (FAMD) at the individual level divided by cluster analysis. In this cluster analysis, age at disease onset and duration of disease symptoms were used as continuous values.

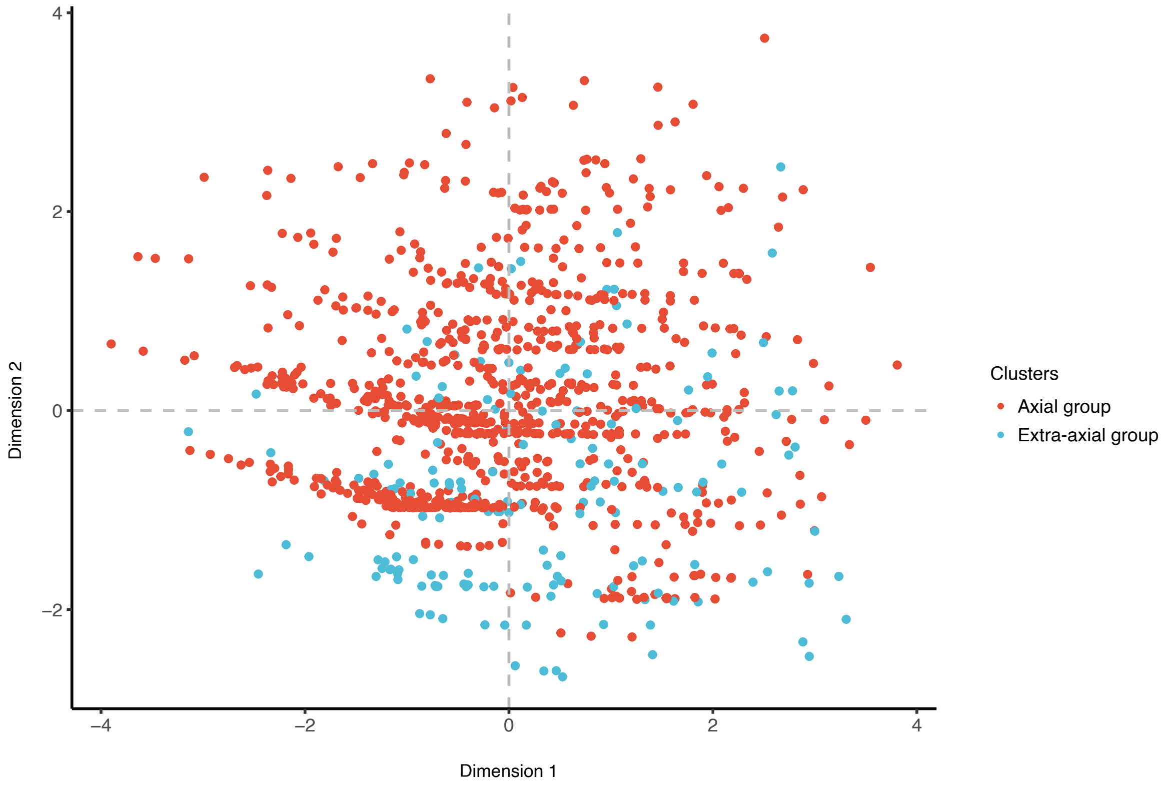

Figure S5. Graph of average silhouette width according to the number of clusters.

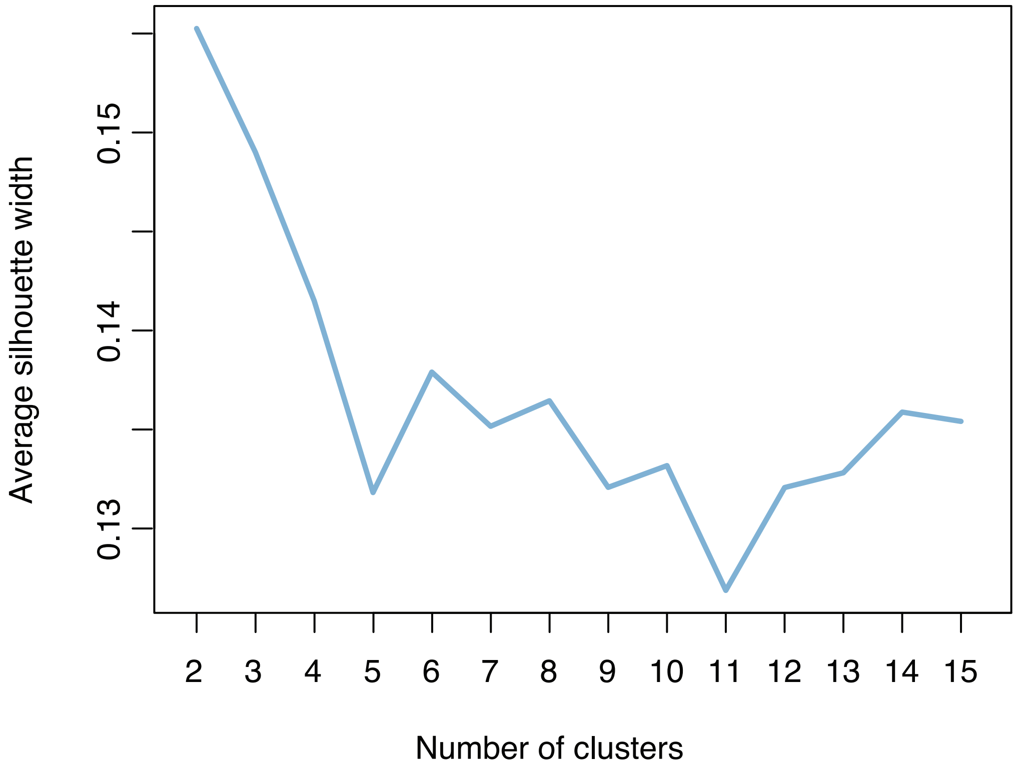

Supplement: Supplementary file 2 — Additional file 2: Figure S1: Results of multiple correspondence analysis (MCA). The first factorial plane with the x-axis and y-axis represents the first and second most important dimensions, respectively. Visualisation of the coordinates of each variable category. Each symbol represents the presence or absence of the variables. Figure S2: Results of sensitivity analyses. Drug survival probabilities of tumour necrosis factor alpha inhibitors (TNFi) in each group: (A) including patients who stopped TNFi for reasons other than inefficacy or adverse event; (B) including patients who stopped TNFi owing to remission. Figure S3: Drug survival probability of tumour necrosis factor alpha inhibitors (TNFi) according to (A) radiographic classification and (B) human leukocyte antigen (HLA)-B27 positivity. Figure S4: Results of factor analysis of mixed data (FAMD) at the individual level divided by cluster analysis. In this cluster analysis, age at disease onset and duration of disease symptoms were used as continuous values. Figure S5: Graph of average silhouette width according to the number of clusters. [file 13075_2021_2647_MOESM2_ESM.pdf]
